# Giant coronary artery aneurysm with a thrombus secondary to Kawasaki disease

**DOI:** 10.4103/0974-2069.41059

**Published:** 2008

**Authors:** Sushant Patil, Salil Shirodkar, Robin J. Pinto, Bharat Dalvi

**Affiliations:** Department of Cardiology, Dr. Balabhai Nanavati Hospital, Mumbai, India

**Keywords:** Coronary artery thrombus, giant aneurysm, Kawasaki's disease

## Abstract

Although coronary artery aneurysms occur in Kawasaki disease, giant aneurysms are rare. We report a very large coronary artery aneurysm, measuring 25 mm and involving left anterior descending artery, in a 2-year-old child with Kawasaki disease. The challenges in management of such a patient have been highlighted.

## CLINICAL SUMMARY

**Figure 1:**
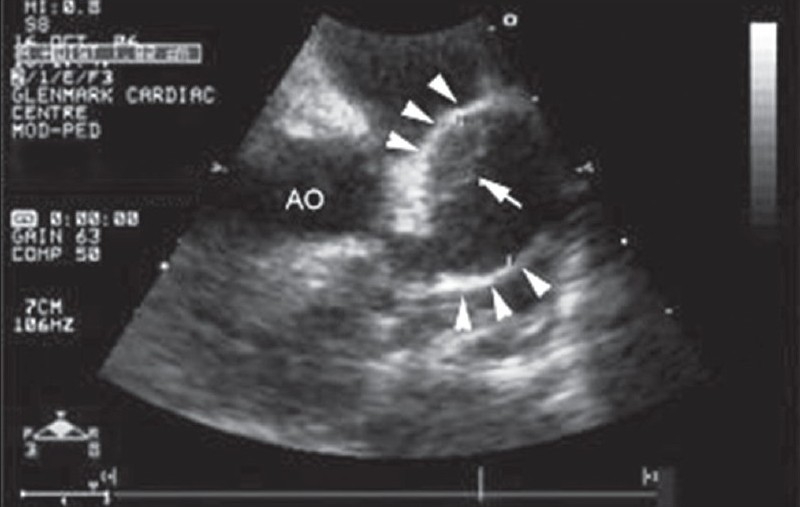
Two-dimensional echocardiography in short-axis view at the level of aortic valve showed a large aneurysm (arrow heads; 18 mm) involving the proximal left anterior descending artery (LAD) with the presence of thrombus (arrow)

**Figure 2:**
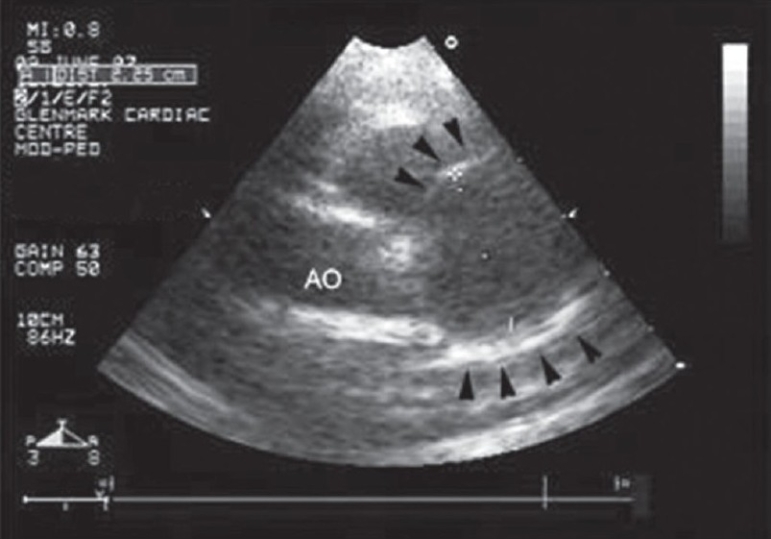
Follow-up echocardiography in short-axis view at the level of aortic valve demonstrating increase in the size of aneurysm (arrow heads) with complete resolution of the thrombus

**Figure 3:**
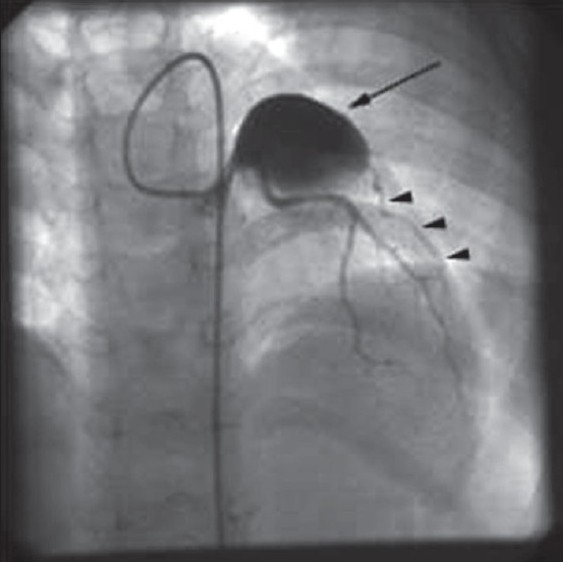
Left coronary angiography in posterior-anterior projection with cranial angulation shows a giant aneurysm (arrow) measuring 25 mm involving the left anterior descending artery (LAD) just after the origin. Note faint opacification of the distal LAD (arrow heads)

**Figure 4:**
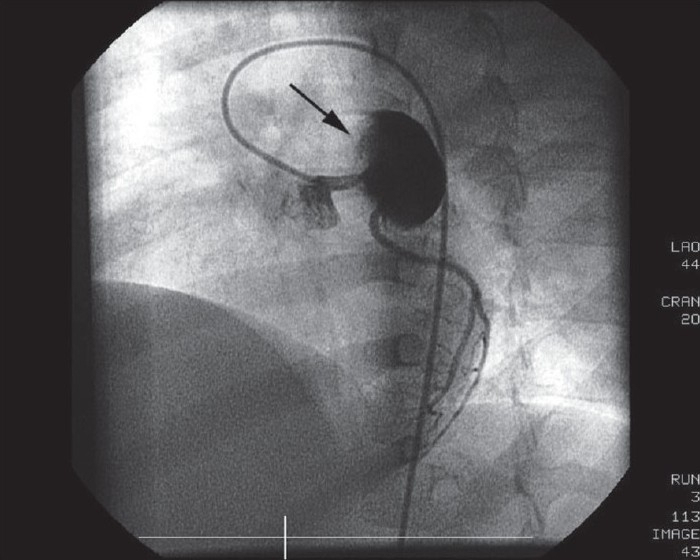
Left coronary angiography in the left anterior oblique projection shows a normal left main coronary artery and a normal non-dominant left circumflex artery. Arrow points toward the giant aneurysm of the LAD

**Figure 5:**
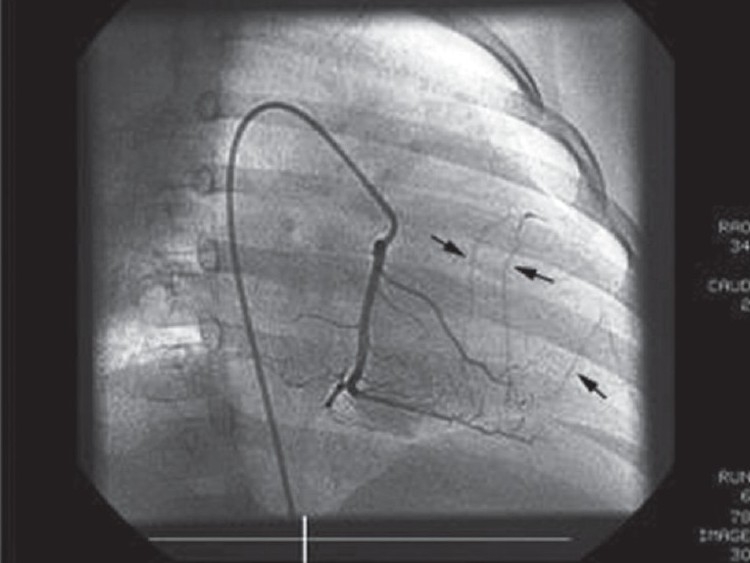
Right coronary angiography in the right anterior oblique view shows a normal dominant right coronary artery with collaterals (arrows) supplied to the left anterior descending artery (LAD)

A 2-year-old girl presented with fever, irritability, and a maculopapular rash of 7-day duration. On examination, she was febrile and had tachycardia, palmar and plantar erythema, cervical lymphadenopathy, bilateral conjunctival injection with congestion of oral mucosa and throat. Her systemic examination was normal. Her hemoglobin was 11.2 g/dl and total white blood cell count was 16,100/mm^3^ with ESR of 60 mm at the end of 1 hour. Her platelets were 4.2 × 10^5^/mm^3^. A diagnosis of Kawasaki disease was made based on her clinical presentation and investigations. She was started on intravenous immunoglobulins (IVIg) and high-dose aspirin, after which she was noted to have symptomatic and clinical improvement. Her echocardiography done on admission revealed a giant aneurysm of proximal left anterior descending artery (LAD) measuring around 18 mm with evidence of a thrombus [Figure 1]. She was started on warfarin and the dose was adjusted to maintain INR of about 2.5. A follow-up echocardiogram, done 2 months later, revealed increase in the size of aneurysm (22 mm) with complete resolution of thrombus [Figure 2]. A subsequent echocardiogram after 6 months did not reveal any thrombus, but the aneurysm size increased to approximately 25 mm. She, however, remained asymptomatic throughout her follow-up period. In view of the progressive increase in the size of the aneurysm, coronary angiography was performed to delineate the coronary anatomy, which may prompt more aggressive therapeutic options. Her coronary angiography revealed a giant aneurysm (25 mm) just after the origin of LAD with swirling of contrast within the aneurysm [Figure 3]. There was a delayed and faint opacification of the distal LAD [Figure 4]. Left main coronary artery (LMCA) and left circumflex coronary artery (LCX) were normal; right coronary artery (RCA) was dominant and normal, supplying collaterals to the LAD [Figure 5].

## DISCUSSION

Cardiovascular sequelae of Kawasaki disease include asymptomatic coronary artery ectasia or aneurysm formation, giant coronary artery aneurysms with thrombosis, myocardial infarction, and sudden death. They have been reported in 15-25% of the untreated children.[1] With treatment using IVIg within first 10 days of the disease, the incidence drops down to about 5%.[2] In those who fail to respond to the initial treatment with IVIg, the administration of steroids is associated with an improvement in symptoms and absence of a significant progression in coronary artery abnormalities.[3] The development of myocarditis, congestive heart failure, pericarditis with pericardial effusion, mitral or aortic insufficiency, and arrhythmias is known to occur during the early course of the disease. Coronary artery dilatation can be seen as early as 4 days after the first appearance of fever. It peaks at approximately 4 weeks after the onset of illness.[4] In our patient, the coronary artery involvement was detected about 7 days after the onset of symptoms.

Aneurysms, which occur during the early phase of Kawasaki disease, are known to involve the proximal segments of the major coronary arteries. The commonest sites of aneurysms in the order of frequency include the proximal LAD as was seen in our patient, proximal RCA, followed by the LMCA, LCX, and finally the distal RCA. The aneurysms are classified as small (<5-mm internal diameter), medium (5-8 mm internal diameter), or giant (>8-mm internal diameter).[5] Approximately 50% of these lesions regress within 5 years. In the majority of those with small coronary artery aneurysms (3-4 mm), regression occurs within 2 years.[6] The risk of aneurysm is increased in patients with fever lasting for more than 16 days, those who have recurrence of fever after an afebrile period of at least 48 h, and those younger than 1 year. Some laboratory values predictive of aneurysm development include low hematocrit levels, thrombocytopenia, and elevated neutrophil/band counts.[7]

The resolution of coronary artery aneurysms depends on the initial size of aneurysm (<5 mm is more likely to resolve than >8 mm), age of the patient at the onset of disease (more likely to resolve in those <1 year age), morphology (fusiform more likely to regress than saccular aneurysm), and location of the aneurysm (distal more likely to involute as compared to the proximal).[8]

All patients with Kawasaki disease should undergo echocardiography on diagnosis and 6-8 weeks after the onset of the disease. Those with giant aneurysms may require a stress test and possibly coronary angiography to identify stenotic lesions.[9] Our patient was too young to perform a stress test. We proceeded with coronary angiography in view of progressive increase in the size of the aneurysm as was evident on serial echocardiography. In our patient, discrete narrowing of LAD was not very well appreciated due to massive aneurysm located just proximal to it. However, a significant reduction in the LAD flow was evident by the poor distal flow associated with delayed filling of the LAD. An extensive collateral supply from the RCA was also a pointer toward significant stenosis of the LAD.

The addition of warfarin to aspirin therapy has been recommended for those with giant aneurysms as was done in this patient. Attempts at excision or plication of the aneurysm have not been successful and have even caused deaths. The arterial graft patency rate in later adult life is still unknown. Percutaneous coronary intervention with placement of a covered stent was not feasible in our patient in view of small vessel size and potential for the artery to grow. In view of these limitations and in the absence of any obvious evidence of myocardial ischemia, it was decided to treat this patient conservatively at the present time. Management of such patients remains a dilemma due to rarity of such giant aneurysms and a very limited data available on the surgical and catheter interventions.
